# Sodæ Sulphis and Hyposulphis in the Treatment of Variola and Varioloid

**Published:** 1868-07

**Authors:** William H. Price

**Affiliations:** Danby, Ill.


ARTICLE XXIII.

### SODÆ SULPHIS AND HYPOSULPHIS IN THE TREATMENT OF VARIOLA AND VARIOLOID.

BY WILLIAM H. PRICE, M.D., Danby, Ill.

Since Prof. Polli's admirable treatise upon the use of the sulphites and hyposulphites in the treatment of zymotic diseases has been laid before the profession, I have been fully determined to test the value of these remedies in the treatment of small-pox, as soon as an opportunity should occur; and since that opportunity has already presented itself, in which my expectations of those remedies have been fully realized, I thought a brief description of the following cases might not prove uninteresting to the many readers of your valuable journal:

CASE I. Jan. 17, 1868, was called to see Patrick B., aged 25, by occupation a daily laborer, and who, as I was informed by the messenger, had been bleeding furiously all the previous night from the right nostril. On my arrival, and after having been introduced to my patient, I immediately recognized the case as being one of discrete variola, which was just entering the stage of suppuration, notwithstanding the patient had been vaccinated while a youth.

On questioning the patient closely, I soon learned that he had been exposed to small-pox in Chicago, and had already passed through the febrile and eruptive stages.

The symptoms of the case were as follows: The whole system was in a state of profuse perspiration; the eruption had extended itself pretty thoroughly over the whole body and extremities, some of these pustules on the chest and neck measuring nearly half an inch in diameter; pulse ranging from 80 to 100; bowels rather constipated; tongue coated; appetite poor; urine natural, though passed in unusual quantities. The epistaxis, which amounted to three or four quarts during the previous night, arrested itself towards morning by a plug of coagulated blood, which I ordered not to be disturbed until the following day, when it came away while the patient was being washed, without any further loss of blood.

The fearful epistaxis, together with the extensive eruption and suppuration, gave the patient a rather deplorable aspect.

I immediately prescribed a saturated solution of equal parts of sulphite and hyposulphite of soda, to be taken in teaspoonful doses every three hours until the bowels were freely opened, when the hyposulphite should be discontinued and the sulphite continued, *ad libitum*, until my next visit. In addition, I ordered a generous diet, with strict cleanliness and free ventilation.

*Jan. 18.* Found the patient very much improved, both physically and mentally. Rest good; head clear; no return of the epistaxis; urine not secreted in such large quantities; pulse 70; bowels open; appetite rather better; with some tendency to perspiration still remaining. I omitted the hyposulphite, but continued the sulphite in the usual large doses.

*Jan. 19.* Patient improving rapidly, with a decided improvement in all the symptoms. The stage of desiccation is now fully inaugurated. The appetite and sleep is now good, and the patient's spirits very much elated. Ordered the sulphite of soda to be continued in large doses. I informed the patient that I would again visit him in three days.

On the second day after my last visit, the patient despatched a messenger to inform me that my services were no longer required, for the reason that he (the patient) was able to be about the house, and was feeling quite well. He, however, continued to take the medicine for several days, after which he called at my office, stating that he was as well as ever, having made a perfect recovery without being marked in the slightest degree.

Case II. Was called, Jan. 30, 1868, to see Mr. Charles P., aged 30, and a lawyer by profession, who was complaining of a severe pain in the head and spinal column, accompanied with slight chills and fever.

On questioning this patient closely concerning his previous history, I was informed that he had been during the years 1865 and 1866 very much exposed to the malarial regions of Kansas and Oregon, and had suffered at that time from a severe attack of ague, from which he thought he had never entirely recovered. On further inquiry, he stated that he had been exposed during the week to severe cold, and more especially during the night of the great fire in Chicago, which, taking into consideration the existing symptoms, was sufficient cause in my mind to give rise to a renewed attack of the old complaint. Having previously used the hyposulphites in cases presenting these symptoms, I accordingly ordered the hyposulphite of soda in 10 gr. doses, in solution, every four or six hours.

*Jan. 31.* Found the patient very much improved, having been relieved entirely of the chills and the pain in the back and head. At this visit, a very slight eruption was noticed upon the forehead, which the patient stated he had noticed on previous occasions after having been attacked by ague. The same treatment was continued.

*Feb. 3.* Found the eruption more fully developed upon the forehead and face, and just making its appearance upon the body and extremities, and presenting all the characteristics of the eruption of varioloid. The patient's bowels being now rather loose, the hyposulphite of soda was discontinued and the sulphite ordered, in large doses, every four hours, together with liberal diet and well-ventilated apartments.

*Feb. 4.* Found the eruption more fully developed upon the body and extremities, with a slight appearance of the stage of suppuration on the forehead and face. The symptoms present at this visit confirmed my diagnosis. All the functions seemed to be regular, and the patient was able to sit up and walk about his room. The sulphite was continued.

*Feb. 6.* Found the patient passing rapidly through the stage of suppuration, and on some portions of the face verging upon the stage of desiccation, without any unfavorable symptoms whatever. The sulphite was continued in the usual large doses. From this time forward, the patient made a rapid recovery, and in a few days made his appearance, without a mark of the disease to be seen.

CASE III. Feb. 16, was called to see Mrs. B., aged 38, the mother of five children, was suffering somewhat from pain in the head and back, and nausea and vomiting, during the evening of the 14th. Feb. 15th. Severe pains in the head and back continued, together with high fever and quick pulse. Feb. 16th, the day on which I first saw the patient, I did not make a positive diagnosis, although there was a slight eruption to be seen on the forehead and arms. Pulse was very quick; bowels rather loose; tongue coated; urine natural in quality and quantity; pain in the back and head very severe. I immediately prescribed sodæ hyposulphis, in from 5 to 10 gr. doses, every three hours. On the evening of the 16th, the bowels were very loose; fever quite high; pulse 110; pains severe in the loins, back, and head. An anodyne was ordered, to relieve pain and to produce sleep, as well as check the bowels, which were now quite loose. The hyposulphite was continued.

*Feb. 17.* The previous night was passed without sleep, and without any amelioration of those severe symptoms already described. Pulse was 120. The hyposulphite was then ordered to be discontinued, and potass. bromide, in saturated solution, to be given in teaspoonful doses every three hours, until the pain should be somewhat relieved and sleep produced. During the evening of the 17th, the patient expressed herself very much better, those severe symptoms being, in a great measure, relieved. Ordered the potass. bromidi to be continued all night.

*Feb. 18.* Patient entirely relieved of pain, after having had a good night's rest. The stage of eruption was now fully inaugurated, and I then was positive that I had a case of semi-confluent small-pox to deal with, and informed the patient and friends accordingly. The potass. bromidi was discontinued, and the sulphite of soda ordered to be given in ℈ doses, every two or three hours. The body to be sponged with tepid or cold water; room to be well ventilated; together with generous diet and plenty of ripe fruits. I must now add that the menses made their appearance, which contributed largely to weaken the patient.

*Feb. 19.* The stage of eruption is fast progressing, being on the face and forehead of the confluent, and on the body of the discrete variety. Appetite is very fair, and patient rests well; pulse 80; bowels regular; very little perspiration. Ordered an infusion of prunus Virginiana to be given with the sulphite.

*Feb. 20.* Stage of eruption seems to be nearly completed. Patient passed a good night; bowels regular; tongue not coated; appetite very good; head a little confused; throat somewhat sore; feet and hands a little swollen; pulse 70. Patient complains to-day of excessive salivation, although she has taken no medicine which would produce such symptoms since the invasion of the disease. The same treatment was ordered to be continued.

*Feb. 21.* Patient seems to be as comfortable as yesterday, excepting the difficulty of deglutition, which is somewhat aggravated. Pulse 75; bowels regular; tinnitus aurium not quite so bad to-day; face considerably more swollen to-day than yesterday; appetite is very fair. Patient's face and forehead is now one solid mass of eruption. The stage of suppuration seems to be now setting in, accompanied with intense fever, and a burning sensation over the surface of the face and head. Menstruation still continues, although not accompanied with so large a flow of menstrual fluid as yesterday.

*Feb. 22.* Stage of suppuration is progressing slowly on some parts of the body, while the eruptive stage does not seem to be quite completed on other parts. The swelling on the face, forehead, hands, and feet seems now to be at its height. Pulse and bowels regular, appetite good, and rest at night better than heretofore. In addition to the sulphite and infusion of wild cherry, which has constituted the principal treatment since the invasion of the disease, a pill of from 3 to 5 grs. of assafœtida is ordered to be taken *ter die*. The patient is allowed to be sponged with whatever fluid substance seems to afford her the most relief; sometimes with cold water, but oftener with sour milk. The menses have now ceased to flow.

*Feb. 23.* Patient is in good spirits. Stage of suppuration is now progressing rapidly, especially upon the body and limbs. The itching and burning sensation upon the face, forehead, and head, which has so much annoyed the patient, is now transferred to the body and limbs. The appetite is good, bowels regular, etc., etc. The same treatment is continued.

*Feb. 24.* Stage of suppuration is now being rapidly completed. The stage of desiccation is making a very slight appearance on the forehead. There is considerable hoarseness, caused by laryngeal and bronchial irritation. The appetite is very good. The patient has not, as yet, shown any symptoms of depression and exhaustion, either in mind or body, but, on the contrary, manifests considerable vitality. The mucous membranes are now very much involved: those of the nose, mouth, larynx, and trachea being the seat of an eruption. The tongue and palate have become covered with vesicles. The throat is quite sore, there being considerable hoarseness and some difficulty of deglutition.

*Feb. 25.* Patient presents symptoms of drowsiness; otherwise appears to be doing well. The urine was examined, and found to contain albumen in a moderate quantity. The swelling of the parotids, face, and eyelids is somewhat subsiding. The lingual, laryngeal, and bronchial irritation is not so great as it was yesterday and the day before. The stage of desiccation is showing itself more clearly upon the forehead and head. Pulse 80, bowels regular, and the urine is passed in the usual quantity.

*Feb. 26.* The patient is apparently doing well. The swelling of the face, eyes, and parotid glands is rapidly subsiding. All of the organic functions seem to be performed as well as they can under the circumstances. The appetite is quite good, and the bowels are open. The patient is allowed to partake freely of ripe fruits and well-cooked vegetables. The stage of desiccation has not set in upon the body and limbs, but is pretty well established upon the face and head. The treatment is now equal parts of the sulphite and hyposulphite of soda, the latter being given in conjunction with the former for the double purpose of keeping the bowels in a soluble condition, and furnishing to the blood a powerful catalytic. The assafœtida pill is continued *ter die*, together with the infusion of the prunus Virginiana.

*Feb. 28.* Patient improving rapidly. The stage of desiccation is pretty well advanced upon the face and head, but just commencing upon the body and limbs. The patient has not, as yet, the slightest symptoms of delirium or convulsions. The appetite is very good; the bowels are very regular; the urine is passed freely and normally, although still containing slight traces of albumen. The hyposulphite is continued, but the sulphite discontinued, in order to insure regularity of the bowels.

*Feb. 29.* Found the patient doing remarkably well. All the functions are regular, and there is every appearance of a tolerable amount of strength and vitality in the system. The stage of desiccation is rapidly progressing. The same treatment is continued.

*March 1.* Found the patient doing well in every respect. The stage of desiccation is pretty well advanced; the crusts are falling off rapidly; the appetite and sleep are good. The urine was to-day examined, and found to contain no albumen. The patient appears to be convalescing without any unfavorable sequelæ whatever. The same treatment continued.

*March 6.* Patient has been doing well since last visit. The crusts have nearly all fallen off, except those upon the arms and lower extremities. All the functions seem to be quite regular; sleep and appetite very fair. The patient complains of weakness in the back and joints, and some general depression, in consequence of which the hyposulphite is discontinued, and tonics are ordered in its stead, together with generous diet.

*March 10.* Patient able to sit up most of the day in a chair. Appetite is very good. Patient complains of being very weak in the joints, especially of the lower extremities. The tonic treatment is continued.

*March 15.* Patient improving very rapidly. From this time forward, the patient continued to improve in strength under the tonic treatment, and in a few days was able to attend to her household duties with as much vigor and energy as ever.

CASE IV. Eugene B., aged 4 years, son of Mrs. B., was complaining March 1st, 1868, of sore throat, with intense pain in the back and head, together with high fever. The hyposulphite of soda was prescribed.

*March 2.* The pain and fever was somewhat relieved. The hyposulphite was continued.

*March 3.* Noticed a slight eruption upon the face and forehead, very much resembling the eruption of measles. The bowels were constipated and pulse very quick. Ordered laxatives and milk diet.

*March 4.* Found the eruption more fully developed, and all the symptoms of a genuine case of discrete variola. The hyposulphite of soda was continued in 5 gr. doses, every two or three hours, and a liberal diet of milk was ordered.

*March 5.* Found the stage of eruption nearly completed. Bowels were somewhat constipated; urine passed freely; appetite and sleep very fair.

*March 6.* The stage of suppuration is now making its appearance upon the body and limbs, as well as upon the head and face. There is one thing remarkable in this case, however, that is worthy of mention, *viz.:* During the first two days of this patient's illness, there was considerable difficulty of deglutition and respiration, as has been already stated, and for the relief of which a narrow strip of flannel, well saturated with kerosene oil, was placed around his throat. At my next visit, these symptoms were very much relieved. After the stage of eruption had been nearly completed, it was noticed by myself and attendants that there was no eruption anywhere to be seen upon the throat, on which the saturated flannel had been placed. Now, whether the application of the kerosene had anything to do with preventing the appearance of the eruption upon the throat and neck, or not, is a question I am unable to decide without further experience. The theory looks rather plausible, however, for the reason that well-formed small-pox pustules are now to be seen scattered over the whole body, except around the patient's neck.

*March 8.* The stage of suppuration is rapidly being completed. All the symptoms at present seem to be very favorable. The hyposulphite of soda is continued.

*March 10.* Patient sleeps well, and has a good appetite. Stage of suppuration is nearly completed. Urine was examined and found to contain no albumen. The same treatment continued.

*March 14.* Patient doing well. The stage of desiccation is now rapidly advancing, without any unfavorable symptoms; has good appetite, and sleeps well; bowels rather constipated, in consequence of partaking too freely of boiled milk. Ordered laxatives. Hyposulphite is continued.

*March 25.* Our little patient has, without any unfavorable or distressing sequelæ, made a rapid and perfect recovery.

Case I, as has already been stated, was contracted in Chicago, and came home to be sick in a family of five in number, *viz.:* the father, mother, and three children. The patient had passed through the first and second stages of the disease, and nearly through the third, before these children, who had been daily exposed, were vaccinated; and yet they escaped from the dreadful malady. From this one case in this family, in a thrifty neighborhood, no other cases originated.

Case II was exposed in Chicago, and came home to expose a loving wife and three little children, who had never been vaccinated until within two days of the father's arrival; and yet these little ones, together with the mother and attendants, entirely escaped from the disease.

Case III, whose family of four children, together with herself, nurse, and husband, had been vaccinated fourteen days previous to the invasion of the disease, was contracted by washing the clothes of Case II. During this patient's illness, the family, cooked, ate, drank, and slept in the same room with the patient; and, strange to say, not one of them took the disease except the little boy, who represents Case IV. The remaining three children, together with the nurse and father were vaccinated at the same time the mother and boy were, and with the same virus. With these four cases, this small epidemic of variola in Danby and vicinity terminated.

Before closing this article, I will add that it is my humble opinion that the sulphites and hyposulphites, as a basis in the treatment of small-pox and zymotic diseases generally, if properly and judiciously used, are superior to any other remedies now known in the profession.

As Prof. Polli says: The sulphites and hyposulphites not only destroy the deteriorating power of the *materies morbi* upon the cerebro-spinal and organic nervous centres; not only decrease the amount of albumen and increase the amount of urea in the urine; but directly destroy the fermento-reproductive power of the poison in the blood, so that the system, if aided by proper nutriment and sustaining treatment, will ultimately eliminate the morbid material, and, in due time, speedily establish convalescence.

